# Supplementary material for: Internal audit quality and accounting information comparability: Evidence from China
Source: PLoS One. 2024 Oct 31;19(10):e0310959. doi: 10.1371/journal.pone.0310959 (PMC11527310; doi:10.1371/journal.pone.0310959)
Supplement: Attachment — Submitted filename: Review_PONE-D-24-12628.docx [file pone.0310959.s002.docx]

**Manuscript title: INTERNAL AUDIT QUALITY AND ACCOUNTING INFORMATION COMPARABILITY: EVIDENCE FROM CHINA**

**Manuscript ID: PONE-D-24-12628**

Originality: Does the paper contain new and significant information adequate to justify publication?

**Answer:**

The introduction needs to be revised. Indeed, the research gap has been clearly stated, however, the key research problem and background of the research are hard to identify. There is no debate about the importance of internal audit quality which could lead to its influence on accounting information comparability. The paper also needs to clearly articulate the value addition of the paper. Try to answer, the “So what?” question then embed it into the manuscript to better enhance the introduction.

Relationship to Literature: Does the paper demonstrate an adequate understanding of the relevant literature in the field and cite an appropriate range of literature sources? Is any significant work ignored?

**Answer:**

Despite cited prior literature related to internal audit quality, this study lacks on discussion on hypothesis development. In addition, theory usage as the baseline in creating hypothesis is not clearly stated. It is better to divide hypothesis development into three parts following this research hypothesis. The chosen setting sample in this research is not well explained.

Methodology: Is the paper's argument built on an appropriate base of theory, concepts, or other ideas? Has the research or equivalent intellectual work on which the paper is based been well designed? Are the methods employed appropriate?

**Answer:**

The manuscript uses appropriate methods to obtain the research aim. Better for the author to mention the number of initial samples first before mentioning several criteria to obtain the final sample, to mention the sample distribution to enhance the story. The sample used for the period of 2017-2022, is not understandable. I recommend providing the reason why this period is used in this research.

Results: Are results presented clearly and analysed appropriately? Do the conclusions adequately tie together the other elements of the paper?

**Answer:**

The discussion about the data analysis result seems missing from the manuscript. I recommend adding discussion in each data analysis result.

Practicality and/or Research implications: Does the paper identify clearly any implications for practice and/or further research? Are these implications consistent with the findings and conclusions of the paper?

**Answer:**

It is better to state the implications explicitly from both a theoretical and a practical perspective to ensure the value addition.

Quality of Communication: Does the paper clearly express its case, measured against the technical language of the field and the expected knowledge of the journal's readership? Has attention been paid to the clarity of expression and readability, such as sentence structure, jargon use, acronyms, etc.

**Answer:**

I barely understand several sentences, so I recommend to do proofread
