# Supplementary material for: Internal audit quality and accounting information comparability: Evidence from China
Source: PLoS One. 2024 Oct 31;19(10):e0310959. doi: 10.1371/journal.pone.0310959 (PMC11527310; doi:10.1371/journal.pone.0310959)
Supplement: Attachment — Submitted filename: Response to Reviewers.docx [file pone.0310959.s003.docx]

Dear Editor and Reviewers,

Thank you for your helpful suggestions. Based on your constructive comments, we re-understand, re-think and strengthen the topic selection, writing logic and core issues of this article. Following your suggestions, we have further revised each part of the paper carefully, including the abstract, introduction, research design, research conclusions and managerial implication sections as follows.

| Response to Reviewer 1 Comments | | |
| --- | --- | --- |
| **1. Summary** |  |  |
| Many thanks for your review of our manuscript. Indeed, as you stated, our manuscript is a pretty good study. At the same time, it contains many drawbacks and needs further revisions. Based on your comments, we have made comments about model and variables, and divided Internal Audit Quality into two groups for analysis. | | |
| **2. Point-by-point response to Comments and Suggestions for Authors** | | |
| **Comments 1:**  Clarify and explain more deeply the theories related to the model and variables in the research model. | | |
| **Response 1**:  We greatly appreciate and fully agree with your suggestions. Indeed, we do not have a detailed explanation of the model and the variables in our manuscript. In accordance with your opinions, we have added the content of this part. Such as:  In order to examine the impact of internal audit on the comparability of accounting information, following the ideas of Peng et al. (2023) [55] and Francis et al. (2014) [18], we have established the following model based on the following reasons: firstly, the OLS model has fewer related constraints, which can effectively construct the correlation between the explanatory variable and the dependent variable. Secondly, by referring to previous literature, the main factors affecting the comparability of corporate accounting information were added to the model, effectively avoiding the interference of relevant factors and enhancing the robustness of the results. Thirdly, we controlled for annual fixed effects and industry fixed effects in the model to avoid the interference of features that do not change over time and industry on the research results. (line 401-410) | | |
| **Comments 2:**  Considering Internal Audit Quality, dividing into two groups with good quality and no quality, the comparison will be clearer. | | |
| **Response 2:**  We attach great importance to the issue you mentioned regarding grouping about Internal Audit Quality. Following your suggestions, we have divided Internal Audit Quality into two groups for analysis in **4.3.2**. Such as:  Reclassifying the internal audit level based on the annual industry median, we define the variable IAQ1. Assigning a value of 1 to samples with IAQ1 greater than or equal to the median, and 0 otherwise. Substituting IAQ with IAQ1 in model (6), the regression results in Column (2) of Table 4 indicate that the IAQ1 coefficient remains significantly positive, and the benchmark regression results exhibit robustness. (line 473-477) | | |

**Finally, thank you for your helpful suggestions. We sincerely hope that we have addressed all concerns in the revision. We look forward to continuing to improve our manuscript with your help.**

| Response to Reviewer 2 Comments | | |
| --- | --- | --- |
| **1. Summary** |  |  |
| Many thanks for your review of our manuscript. You pointed out essential links between internal control and internal audit, concentrated ownership structure in China, and empirical design in the previous manuscript. We have made substantial modifications to theoretical hypotheses and empirical design based on your comments. We believe that these modifications will help to improve the quality of our manuscript. | | |
| **2. Point-by-point response to Comments and Suggestions for Authors** | | |
| **Comments 1:**  Given the essential links between internal control and internal audit, authors might also want to explicitly illustrate how this paper extends the discussions in the literature, which is highly desirable for the paper to sharpen the contribution. | | |
| **Response 1**:  We greatly appreciate and fully agree with your suggestions. It is true that we didn't compare the relationship between internal control and internal audit of our manuscript should be expanded. According to your opinions, we have supplemented related content of this study in “**1.Introduction**” and “**5.1 Internal audit quality, internal control level and accounting comparability**”. The specific contents are as follows are as follows:  **(1)** Conversely, internal control faces challenges due to information asymmetry and increased transaction costs, hindering shareholders or ultimate controllers from obtaining effective information for direct management supervision. At this juncture, internal control lacks substantive independence. In contrast, internal audit, with its dual functions of confirmation and consultation, is better positioned to improve the quality of accounting information. Enhanced professional personnel, increased project activities, and effective feedback mechanisms within internal audit institutions are instrumental in bolstering their independence and authority for effective management supervision, thereby enhancing the quality of accounting information. Moreover, leveraging the expertise of audit committees to provide advisory functions further enhances the quality of accounting information. (line 43-53)  **(2)** Although Guo and Guo (2021) [28] have found that the mechanism by which internal control improves information comparability is also to reduce agency costs and improve information quality, we believe that this does not hinder the effectiveness of internal audit functions, as internal audit and internal control are interdependent and coupled [29]. On the one hand, high-quality internal auditing can improve the soundness and effectiveness of internal controls. On the other hand, sound internal controls can provide a solid foundation for internal auditing. This is because a well-designed and operational internal control system can provide a good foundation for risk control and information credibility, providing a reliable working foundation for internal audit. It also puts forward direction and requirements in determining the scope and focus of auditing, selecting audit procedures, methods, and other aspects. Therefore, the higher the internal quality level, the more conducive it is to the performance of the internal audit function. (line 113-124)  **(3)** Meanwhile, we verified the impact mechanism of internal audit on comparability from multiple perspectives such as internal control and industry competition, and provides evidence of the interaction between internal audit and internal control. (line 135-138)  **(4)** Previous studies have shown that robust internal controls, by closely monitoring managerial actions, can reduce earnings management in a company [70] and improve the quality of accounting information disclosure [1, 71]. Therefore, the mechanism for enhancing accounting information comparability through internal control closely mirrors that of internal audit. Due to the interdependence between internal audit and internal control, as they intersect and interact [29], higher internal control quality enhances the effectiveness of internal audit functions, thereby facilitating the improvement of accounting information comparability.  To investigate the moderating effect of internal control, we utilized the DIB internal control rating index, denoted as InterCon, which reflects higher internal control quality. Ratings from AAA to D were assigned values from 8 to 1, respectively, and the interaction term InterCon×IAQ was included in model (6) for regression analysis. The results in column (1) of Table 8 show a significantly positive coefficient for the interaction term, indicating that internal control quality reinforces the positive relationship between internal audit and accounting information comparability. (line 615-629) | | |
| **Comments 2:**  Authors suggest that internal audit can increase monitoring on management, which then improves information comparability. However, given the concentrated ownership structure in China, it is likely that the internal audit functions in different channels as those in markets with dispersed ownership structures. Authors can incorporate the unique features of Chinese firms in the theoretical analyses. | | |
| **Response 2:**  We attach great importance to the issue you mentioned regarding concentrated ownership structure in China. It is indeed that while internal audit helps mitigate agency problems between management and owners, it does not address the agency issues between major shareholders and small and medium-sized shareholders. In light of your feedback, we added relevant tests in “**5.3 Internal audit quality, capital appropriation by major shareholders and accounting comparability**”. The specific contents are as follows are as follows:  As an emerging economic country, China's corporate equity is relatively concentrated, with the conflict of interests between major shareholders and small and medium-sized shareholders emerging as a predominant agency issue [74,75]. The self-interest behavior of major shareholders can negatively impact economic transparency [76,77], thereby potentially influencing the comparability of accounting information. The severity of the agency problem between major shareholders and small and medium-sized shareholders determines the extent of major shareholders' encroachment on the interests of smaller shareholders. Consequently, major shareholders are more likely to exploit their power to undermine the quality of internal auditing and restrict its role in enhancing information transparency.  To examine the influence of major shareholders on the relationship between internal audit and the comparability of accounting information, we utilize the capital occupancy rate of major shareholders, denoted as Occupy [78], to measure the agency cost between major shareholders and small and medium-sized shareholders. This is calculated using the ratio of other receivables to year-end total assets. A higher capital occupancy rate of major shareholders indicates greater agency costs. Examining the results of the interaction term (Occupy×IAQ), we observe a significantly negative coefficient. This suggests that in an environment characterized by high agency problems among major shareholders, the positive impact of internal audit level on comparability is diminished. Comparing these results with previous findings, it appears that while internal audit helps mitigate agency problems between management and owners, it does not address the agency issues between major shareholders and small and medium-sized shareholders. Furthermore, this demonstrates the significant control exerted by major shareholders in China. (line 662-683) | | |
| **Comments 3-1:**  Why does the sample period start from 2007? Why not cluster standard errors at the firm level in the baseline model? Authors may want to explain the sample selection. | | |
| **Response 3-1:**  We are very sorry that we did not explain the issue of sample selection period and cluster standard error. We attach great importance to this issue and explain the above problem. We explain the reasons for the sample period selection, also explain the reasons for the industry-level cluster, and perform the company-level cluster analysis in the robustness test.  **(1) As for sample period**, due to the international convergence of accounting standards in China in 2007, we selected A-share listed companies in Shanghai and Shenzhen from 2007 to 2022 as the research sample. (line 294-296)  **(2)** **As for industry-level cluster**, cluster analysis at the industry level is used in the basic regression for the following reasons: First, the comparability of accounting information reflects the similarity or similarity of the same type of accounting items between different enterprises. Therefore, compared with the company level, the industry level is also an important factor affecting the distribution of random disturbance terms. Second, relevant literature research shows that factors at the industry level, such as the degree of industry competition, industry attributes and other endogenous factors have a direct impact on the comparability of accounting information. (line 417-424)  **(3)** **As for firm-level cluster**, in order to avoid the interference of company-level factors on the research results, we further adopted company-level cluster analysis to conduct robustness test. The specific results are shown in column (5) of Table 4. The results show that the coefficient of variable IAQ is significantly positive at the 5% level, indicating that the correlation between internal audit and accounting information comparability remains notably positive on the basis of the company-level cluster analysis, thus further verifying the robustness of the basic regression results. (line 493-499) | | |

**Comments 3-2:**

The authors used a consolidated measure of internal audit quality based on the affiliation model, the responsibility scope, the dual position of chairman and CEO, and whether the firm is audited by a Top 10 Audit Firm. As those dimensions are not similarly constructed by using dummy variables, authors can examine the impact of each dimension separately. This can also help explore the heterogeneous effects among them.

**Response 3-2:**

We greatly appreciate and fully agree with your suggestions. Indeed, it is interesting to explore the heterogeneous effects among those dimensions. Following your suggestions, we conducted in-depth research based on the four dimensions of constructing IAQ, to identify which factors are the key driving factors affecting the comparability of accounting information. The specific contents are as follows are as follows:

Furthermore, this article attempts to conduct in-depth research based on the four dimensions of constructing IAQ, in order to identify which factors are the key driving factors affecting the comparability of accounting information. The specific results are listed in columns (4) - (7), and the results in column (4) indicate that the coefficient of IAModel is significantly positive at the 1% level, indicating that the membership mode of the internal audit department is a key factor affecting comparability in enterprises. Similarly, the coefficient of variable IAduty is also significantly positive at the 1% level, indicating that the larger the scope of internal audit responsibilities, the more conducive it is to improving comparability. However, in contrast, the results in columns (6) - (7) indicate that dual roles and firm selection are not key factors affecting the quality of internal auditing in listed companies. (line450-460)

**Comments 3-3:**

To ensure the robustness of the results, authors can also use other measures of internal audit quality. For example, the factors include the effectiveness of the audit committee proxied by the number of audit meetings, committee size, and background of committee members (e.g., financial and accounting expertise).

**Response 3-3:**

We attach great importance to your question about other measures of internal audit quality. According to your opinions, the number of annual meetings of the audit committee of listed companies (Commission) is used as a proxy variable for the quality of internal audit of listed companies. The specific explanations are as follows:

**4.3.4 Changing the** **independent variable.** In order to avoid the interference of internal audit level measurement bias on research results, explanatory variables are replaced. Specifically, the number of annual meetings of the audit committee of listed companies (Commission) is used as a proxy variable for the quality of internal audit of listed companies. The larger the indicator, the higher the quality of internal audit of listed companies. The specific results are shown in column (4) of Table 4, where Commission is significantly positive at the 5% level, indicating that the more audit committee meetings there are, the stronger the comparability of accounting information of listed companies, indicating that the quality of internal audit can improve comparability. (line484-492)

**Comments 3-4:**

Authors shall recheck the data and results. It seems odd that the coefficient is significant at 10% when t=1.69 in Table 6 while it is insignificant when t=1.71 in Table 3. Given the confusing notations, it is hard to make conclusions from the results.

**Response 3-4:**

We are very sorry that the significance of some regression coefficients is odd. This is because the significance of T-value is affected by sample size and degree of freedom. For example, smaller sample sizes or lower degrees of freedom may require larger T-values to reach the significance level. Therefore, the specific judgment of whether the T-value is significant also needs to be combined with the actual research problem, sample size, freedom and other factors for comprehensive consideration. To avoid the ambiguity, we have replaced T-value with P-value to help readers understand the results correctly. Thank you for your careful review and help us optimize the details.

**Comments 3-5:**

For the subsample analyses, authors shall report the results of the between-group difference tests.

**Response 3-5:**

We greatly appreciate and fully agree with your suggestions. In order to explain the differences between the groups and unify the analysis methods, we analyzed the moderating effects of the four variables (InterCon, CR4, Occupy, Tech) by introducing cross-multiplicative terms in “5. Further analysis”. (line705)

**Comments 3-6:**

Are instruments the same for the Heckman and the 2SLS tests? It seems the target company is excluded in calculating the average mean in 2SLS while it is not when conducting the Heckman two-stage estimation. Authors may want to elaborate on the calculation of instruments.

**Response 3-6:**

Thank you for your careful review. We are very sorry that we didn't clarify the instrumental variables. The instrumental variables are actually the same in both Heckman and the 2SLS tests. However, we had some problems with the application of Heckman test. This is because we used the full sample and there was no sample selection bias caused by the inability to observe internal audit information, so we deleted this test.

**Comments 4:**

There are errors in writing. For example, the authors state that “internal control quality attenuates the positive association between internal audit and accounting information comparability” (pp.37) while the coefficient of the interaction term InterCon× IAQ is positive (0.023), and the authors conclude that “internal control quality fortifies the positive correlation between internal audit and accounting information comparability” (pp.40). That is, the interpretations are conflicting themselves. I would suggest authors have professional editing services to address the writing issue.

**Response 4:**

We are very sorry for the errors in writing. Thanks for your careful review. We have proofread and corrected the error. The modified content is as follows:

The results in column (1) of Table 8 show a significantly positive coefficient for the interaction term, indicating that internal control quality r**einforces** the positive relationship between internal audit and accounting information comparability.

**Finally, thank you for your helpful suggestions. We sincerely hope that we have addressed all concerns in the revision. We look forward to continuing to improve our manuscript with your help.**

| Response to Reviewer 3 Comments | | |
| --- | --- | --- |
| **1. Summary** |  |  |
| We are grateful for your acknowledgement of our manuscript. Our manuscript contains many drawbacks and needs further revisions. Based on your comments, we have made corrections to the introduction, hypothesis development, research design, and writing to improve the quality of our manuscript. | | |
| **2. Point-by-point response to Comments and Suggestions for Authors** | | |
| **Comments 1:**  The introduction needs to be revised. Indeed, the research gap has been clearly stated, however, the key research problem and background of the research are hard to identify. There is no debate about the importance of internal audit quality which could lead to its influence on accounting information comparability. The paper also needs to clearly articulate the value addition of the paper. Try to answer, the “So what?” question then embed it into the manuscript to better enhance the introduction. | | |
| **Response 1**:  We attach great importance to your question about the introduction. It is true that our introduction is deeply flawed. According to your suggestion, we have supplemented the research background, key research problem, the relationship between internal control and internal audit, China's real problems and the significance of enhancing the comparability of accounting information in the introduction. **The revised introduction is as follows：**  The quality of accounting information in enterprises can alleviate the information asymmetry between internal and external investors, and plays a pivotal role in enhancing the efficiency of capital market resource allocation and safeguarding investor interests. Agency conflicts between owners and managers are important factors affecting the quality of accounting information [1,2]. Previous studies have shown that internal control [3,4], internal audit [5-7], and external audit [8,9] are three effective mechanisms for reducing agency costs and improving the quality of accounting information. Despite the gradual improvement in the Chinese capital market system and legal environment, along with notable advancements in internal governance in enterprises, instances of accounting information violations persist due to inadequate internal oversight. For example, the illegal information disclosure by Changsheng Biotechnology^[1]^, the financial fraud by Kangdexin^[2]^, and the stock price manipulation by Guangzhou Anzhou^[3]^ have all reduced the usefulness of information decision-making and are not conducive to investors making correct investment decisions.  While both external audit and internal control contribute to enhancing the quality of accounting information, internal audit possesses distinct advantages. Firstly, internal auditing, unlike external auditing, comprehensively assesses all management systems within a company and gathers audit evidence through informal methods. Additionally, internal audit staff are primarily internal employees of the enterprise, facilitating easier trust and support from audited personnel. Consequently, internal audit is more likely to exert governance effects and enhance information quality compared to external audit. Conversely, internal control faces challenges due to information asymmetry and increased transaction costs, hindering shareholders or ultimate controllers from obtaining effective information for direct management supervision. At this juncture, internal control lacks substantive independence. In contrast, internal audit, with its dual functions of confirmation and consultation, is better positioned to improve the quality of accounting information. Enhanced professional personnel, increased project activities, and effective feedback mechanisms within internal audit institutions are instrumental in bolstering their independence and authority for effective management supervision, thereby enhancing the quality of accounting information. Moreover, leveraging the expertise of audit committees to provide advisory functions further enhances the quality of accounting information. Hence, re-evaluating the internal audit function and improving accounting information quality are imperative.  Comparability is an important characteristic of accounting information quality, which can reduce the cost of debt financing for enterprises [10,11] and reduce the risk of stock price collapse [12]. Compared to accounting information quality, such as prudence and timeliness, which only reflect the company's own characteristics, comparability can provide incremental information about the industry in which the company operates, reducing the cost of information collection for report users [13]. Firstly, comparability is a key indicator that reveals the unique differences between a company and its competitors in the same industry. It helps investors identify the unique risks of the company and can also assist accounting information users in directly comparing the financial status differences of companies in the same industry. Secondly, comparability can present common characteristics of the industry in which the company operates, which helps clients to excavate and predict the accounting information of the target company by comparing the financial reports of other companies in the same industry [15]. Finally, comparability of accounting information is beneficial for companies in the same industry to enhance their competitiveness through mutual learning, thereby building a more stable and resilient supply chain [16]. Therefore, exploring how to improve the comparability of accounting information is of positive significance for enhancing the decision-making usefulness of external stakeholders [17] and promoting the optimal allocation efficiency of economic resources. Previous studies have found that auditors using unique audit methods and styles can affect accounting information comparability [18-23]. However, due to the availability of data, there has been no research on how internal audit affects the comparability of accounting information as an internal supervision mechanism. From the perspective of the function of internal audit, effective implementation of internal audit can timely detect and correct fraudulent behavior in financial statements [24], and constrain management's improper selection of accounting policies [25]. Therefore, promoting the internal audit function can provide support for enterprises to effectively improve the comparability of accounting information.  However, there is controversy over the impact of internal auditing on the quality of accounting information. Based on agency theory, some studies suggest that internal auditing can alleviate the principal-agent problem, reduce the level of earnings management, and improve the quality of accounting information by monitoring the self-interest behavior of executives [5-7]. However, the executive rights theory suggests that the promotion and other incentives of the head of the internal audit department are constrained by the company's management, and the internal audit department may not be able to maintain independence and play a governance role in the self-interest behavior of the management, ultimately failing to improve the quality of accounting information [26]. Theoretical debates and practical needs require in-depth research based on Chinese listed companies.  There are two main reasons why we focus on the Chinese capital market. On the one hand, as the world's largest emerging market, China's legal and external market supervision mechanisms are not yet sound. Studying the impact of internal auditing on the comparability of accounting information at this time can help us better understand the benefits of strengthening corporate internal governance supervision mechanisms in emerging capital markets. On the other hand, the accounting information quality of Chinese listed companies varies, and the effectiveness of the capital market needs to be improved. The improvement of comparability can improve the information environment of enterprises and reduce the degree of information asymmetry, thereby safeguarding the interests of stakeholders such as investors and creditors, and optimizing resource allocation. Therefore, studying the factors influencing comparability is of great significance for shaping a favorable information environment in emerging market countries.  Therefore, based on the data of Chinese A-share listed companies from 2007 to 2022, this article studies the impact of internal audit on the comparability of accounting information. Firstly, we found that internal audit can improve the comparability of accounting information. Secondly, we investigated the channels through which internal audit affects comparability and found that internal audit improves comparability by reducing agency costs and enhancing financial information transparency. Finally, the moderating effect test proves that the quality of internal control and the high-tech industry will strengthen the positive relationship between internal audit and comparability, while the degree of industry competition and the level of large shareholders' capital occupation will weaken the positive relationship.  Although Guo and Guo (2021) [28] have found that the mechanism by which internal control improves information comparability is also to reduce agency costs and improve information quality, we believe that this does not hinder the effectiveness of internal audit functions, as internal audit and internal control are interdependent and coupled [29]. On the one hand, high-quality internal auditing can improve the soundness and effectiveness of internal controls. On the other hand, sound internal controls can provide a solid foundation for internal auditing. This is because a well-designed and operational internal control system can provide a good foundation for risk control and information credibility, providing a reliable working foundation for internal audit. It also puts forward direction and requirements in determining the scope and focus of auditing, selecting audit procedures, methods, and other aspects. Therefore, the higher the internal quality level, the more conducive it is to the performance of the internal audit function.  The research contribution includes two aspects: theoretical contribution and practical contribution. In terms of theoretical contribution, on the one hand, we have clarified the controversy surrounding the role of existing theories in internal auditing. The agency theory believes that internal audit is an important mechanism for improving accounting information supervision [5,6], while the management rights theory suggests that the internal audit department may collude with management [26]. The above theoretical contradictions need to be clarified. This article is based on the perspective of comparability of accounting information, opening up the black box of the mechanism of internal audit on comparability of accounting information. It is found that internal audit can improve comparability of accounting information by reducing agency costs and improving financial information transparency, providing empirical evidence for theoretical debates. Meanwhile, we verified the impact mechanism of internal audit on comparability from multiple perspectives such as internal control and industry competition, and provides evidence of the interaction between internal audit and internal control. On the other hand, our research enriches the relevant research on the factors affecting the comparability of accounting information at the micro enterprise level. Previous literature mainly studied the impact of external auditors using unique audit methods and styles [18-23] on comparability, but they overlooked the strengthening effect of micro level governance tools of internal audit companies on comparability. Compared to external auditing, internal auditing can examine all management systems of a company, and informal methods can be used to collect audit evidence, affecting the behavior of corporate executives and thus affecting the comparability of accounting information. This study can make up for this deficiency and deepen the understanding of the internal audit supervision function.  In terms of practical contribution, on the one hand, this article helps enterprises improve the construction of internal auditing. Internal audit plays an important role in alleviating agency issues and improving the quality of financial information in enterprises. Therefore, enterprises can further improve their internal audit system, such as strengthening the degree of management's emphasis on the design of internal audit systems, and enhancing the independence and authority of the internal audit department. On the other hand, this article helps to improve the level of internal governance in enterprises. Suppressing agency costs and improving financial information transparency are important paths to improve the comparability of accounting information. Therefore, enterprises should reasonably guide managers to reduce agency problems, which is the key enterprises’ benign development. In addition, our research helps to improve the comparability of corporate accounting information, thereby safeguarding the interests of stakeholders such as investors and creditors, and improving the efficiency of investor decision-making.  The subsequent sections are organized as follows: the second segment comprises a comprehensive literature review and the formulation of hypotheses. The third segment delineates the research design. The fourth segment presents empirical results. The fifth segment undertakes further analysis and the final segment encapsulates the conclusion. (line 24-160)  **We believe your comments can help us improve the integrity and logic of the introduction.** | | |
| **Comments 2:**  Despite cited prior literature related to internal audit quality, this study lacks on discussion on hypothesis development. In addition, theory usage as the baseline in creating hypothesis is not clearly stated. It is better to divide hypothesis development into three parts following this research hypothesis. The chosen setting sample in this research is not well explained. | | |
| **Response 2:**  We greatly appreciate and fully agree with your suggestions. Indeed, our study lacked on discussion on hypothesis development and unstated theory. In accordance with your opinions, we have divided hypothesis development into three parts, stated agency theory, and added the hypothetical model figure (line 291). The revised hypothesis are shown below:  **2.4.1 Internal audit level and comparability of accounting information.** Bath et al. (2012) [17] pointed out that external legal environment, accounting standards, and the interpretation and implementation of accounting standards by financial personnel can affect the quality of disclosed accounting information. According to agency theory, the current accounting standards give enterprises a certain degree of autonomy, and the management, as the direct responsible person, has a direct impact on the accounting selection, recognition, measurement, and other aspects of the enterprise. Therefore, whether there is self-interest in the selection of accounting policies and methods by management becomes the most direct factor affecting the comparability of accounting information. When management engages in opportunistic behavior, they use the given rights and space to choose accounting treatment policies and methods that are most conducive to completing transactions, and manipulate the reporting process of accounting information to reduce its comparability. Previous studies have shown that internal audit, as the main supervisory mechanism for corporate governance [36], can to some extent prevent opportunistic behavior by management [24], alleviate agency conflicts [25], and improve financial information transparency [5-7]. Therefore, we believe that internal auditing can improve comparability by alleviating agency conflicts and improving information quality. Based on the above analysis, this article proposes the following hypothesis:  **H1: Internal audit could improve comparability of accounting information. Specifically, higher levels of internal audit correlate with heightened accounting information comparability.**  **2.4.2 The mechanism of agency problems.** According to the agency theory, internal auditing can suppress the self-interest and opportunistic behavior of executives, providing supervision and management support for improving the comparability of accounting information. The rapid development of the digital economy has led traditional enterprises to face a fierce market competition environment, which is easily affected by funding and liquidity risks, leading to a decline in profitability and generating strong earnings pressure. In order to cope with this kind of earnings pressure, companies that engage in earnings manipulation may choose unreasonable accounting policies to increase accounting profits, resulting in significant differences in accounting procedures and methods used by management who have not engaged in earnings manipulation when accounting for the same economic transactions, thereby reducing comparability. The implementation of internal audit functions can effectively reduce management's earnings manipulation behavior in accounting policy selection [25]. Internal audit achieves effective supervision of managers through its supervisory function [36], which can reduce the first type of agency costs [58], suppress management's self-interest and opportunistic behavior [37], reduce management's discretion in accounting policy choices, and thereby improve comparability. Specifically, internal audit uses systematic and standardized methods to review and evaluate the appropriateness and effectiveness of the management's manipulation of financial statement numbers in collaboration with other functional personnel, increasing the likelihood of management's arbitrary changes in accounting policies being detected, deterring the self-interest and opportunistic behavior of executives, enhancing management's prudence in accounting estimates and policy choices, thereby ensuring consistency in accounting policy choices, and ultimately improving comparability. Based on the above analysis, this article proposes the following hypothesis:  **H2: Internal audit could improve comparability of accounting information by mitigating agency costs.**  **2.4.3 The mechanism of financial information transparency**. Internal auditing can suppress the level of earnings management in enterprises [25], improve the quality of financial information [5-7, 31-33], reduce the drawbacks caused by information asymmetry [34], and create a good information environment for improving the comparability of accounting information. Comparability is an important source of information for information users to make decisions [17], which can reduce the cost of information collection and processing, improve the usefulness of decision-making, and thus protect their legitimate interests. However, in practice, due to information asymmetry between information producers and users, information producers rely on their information advantages and discretion in implementing accounting standards to interfere with the production and disclosure of accounting information, thereby reducing comparability. As Zhou and Yang (2018) [59] found in their research, insiders with information advantages who hide internal transaction behavior will reduce comparability. De Franco (2011) [13] also found that the more transparent the information environment of a company, the higher the comparability of accounting information. It can be seen that improving the information environment of enterprises helps to enhance comparability. Internal audit has a special comparative evaluation function, which can improve the internal information environment of enterprises, enhance the quality of financial information [5-7], correct erroneous information in financial statements, promote the release of true financial data, and ensure the production of high-quality comparable accounting information. At the same time, as the transparency of financial information increases, the likelihood of executives using complex earnings management to conceal bad news decreases. This invisibly weakens the inherent motivation of management to selectively disclose information, increases their prudence in accounting estimates and policy choices, and ensures consistency in accounting policy choices, thereby improving comparability. Based on the above analysis, this article proposes the following hypothesis:  **H3: Internal audit could improve comparability of accounting information by enhancing financial information transparency.** (line 215-289)  **We believe your comments can help us improve the logic of our hypothesis.** | | |
| **Comments 3:**  The manuscript uses appropriate methods to obtain the research aim. Better for the author to mention the number of initial samples first before mentioning several criteria to obtain the final sample, to mention the sample distribution to enhance the story. The sample used for the period of 2017-2022, is not understandable. I recommend providing the reason why this period is used in this research. | | |
| **Response 3:**  We are very sorry that the previous manuscript did not provide more explanations for the sample. Based on your suggestions, we have detailed description of sample period and quantity.  (1) **Sample period**. Due to the international convergence of accounting standards in China in 2007, this study selected A-share listed companies in Shanghai and Shenzhen from 2007 to 2022 as the research sample. (line 295-296)  (2) **Sample quantity**. On the basis of the initial sample (**47698**), the following samples were excluded: (1) The sample of financial listed companies (**1416**) was excluded because their asset structure and profit model are different from general enterprises; (2) Excluding the sample of listed companies with trading status ST, ST *, and PT (**2747**) due to the heterogeneity of their financial status; (3) Exclude a sample of **4426** listed companies that have undergone mergers and acquisitions or changes in their main business, as this would result in changes in performance; (4) Exclude a sample of **409** companies that have been listed for less than a year, as they may be affected by IPO effects; (5) Exclude a sample of **6961** listed companies that have issued both B-shares and H-shares simultaneously, as there may be differences in overseas legal regulation compared to domestic ones; (6) Exclude a sample of **13510** listed companies with incomplete data. A total of **18229** valid samples (**47698-1416-2747-4426-409-6961-13510**) were obtained in the end. (line 298-309) | | |

**Comments 4:**

The discussion about the data analysis result seems missing from the manuscript. I recommend adding discussion in each data analysis result.

**Response 4:**

We greatly appreciate and fully agree with your suggestions. It is true that some data analysis result seems missing. Based on your suggestions, we added descriptions of some of the data, such as:

Table 2 presents the descriptive statistical results of the main variables, reflecting the following main descriptive statistical characteristics: firstly, the average value of the dependent variable (CompAcct) is (-1.28), indicating that there is still significant improvement space in the comparability of accounting information among the sample companies in terms of average significance. Meanwhile, CompAcct fluctuates between (-4.24) and (-0.41), indicating significant differences in the comparability of accounting information among the sample companies. Secondly, the explanatory variable IAQ fluctuates between 0.33 and 4, with a variance of 0.8, reflecting significant differences in the internal audit level of the sample companies. Thirdly, the descriptive statistical characteristics of the relevant control variables are basically consistent with the characteristics of the references. The above features provide an effective sample basis for further research. (line 428-438)

Table 3 reports the regression results of the impact of internal audit on the comparability of accounting information. Column (1) shows the regression results without any control variables, and the coefficient of CompAcct is 0.054, showing a significant positive correlation at the 1% level. Column (2) shows the fixed effects regression results that only include industry and year. The CompAcct coefficient is 0.035, which has significant statistical significance at the 1% level. Column (3) displays the regression results for all control variables. The CompAcct coefficient is 0.020, showing a significant positive correlation at the 5% level. The results indicate that internal auditing can improve comparability. This result preliminarily confirms hypothesis H1. (line 441-449)

Furthermore, this article attempts to conduct in-depth research based on the four dimensions of constructing IAQ, in order to identify which factors are the key driving factors affecting the comparability of accounting information. The specific results are listed in columns (4) - (7), and the results in column (4) indicate that the coefficient of IAModel is significantly positive at the 1% level, indicating that the membership mode of the internal audit department is a key factor affecting comparability in enterprises. Similarly, the coefficient of variable IAduty is also significantly positive at the 1% level, indicating that the larger the scope of internal audit responsibilities, the more conducive it is to improving comparability. However, in contrast, the results in columns (6) - (7) indicate that dual roles and firm selection are not key factors affecting the quality of internal auditing in listed companies. (line 450-460)

**Comments 5:**

It is better to state the implications explicitly from both a theoretical and a practical perspective to ensure the value addition.

**Response 5:**

We attach great importance to your suggestion about implications. Based on your feedback, we have divided the research contribution into theoretical contribution and practical contribution. The specific explanations are as follows:

**The research** **contribution includes two aspects: theoretical contribution and practical contribution**. **In terms of theoretical contribution**, on the one hand, we have clarified the controversy surrounding the role of existing theories in internal auditing. The agency theory believes that internal audit is an important mechanism for improving accounting information supervision [5,6], while the management rights theory suggests that the internal audit department may collude with management [26]. The above theoretical contradictions need to be clarified. This article is based on the perspective of comparability of accounting information, opening up the black box of the mechanism of internal audit on comparability of accounting information. It is found that internal audit can improve comparability of accounting information by reducing agency costs and improving financial information transparency, providing empirical evidence for theoretical debates. Meanwhile, we verified the impact mechanism of internal audit on comparability from multiple perspectives such as internal control and industry competition, and provides evidence of the interaction between internal audit and internal control. On the other hand, our research enriches the relevant research on the factors affecting the comparability of accounting information at the micro enterprise level. Previous literature mainly studied the impact of external auditors using unique audit methods and styles [18-23] on comparability, but they overlooked the strengthening effect of micro level governance tools of internal audit companies on comparability. Compared to external auditing, internal auditing can examine all management systems of a company, and informal methods can be used to collect audit evidence, affecting the behavior of corporate executives and thus affecting the comparability of accounting information. This study can make up for this deficiency and deepen the understanding of the internal audit supervision function.

**In terms of practical contribution**, on the one hand, this article helps enterprises improve the construction of internal auditing. Internal audit plays an important role in alleviating agency issues and improving the quality of financial information in enterprises. Therefore, enterprises can further improve their internal audit system, such as strengthening the degree of management's emphasis on the design of internal audit systems, and enhancing the independence and authority of the internal audit department. On the other hand, this article helps to improve the level of internal governance in enterprises. Suppressing agency costs and improving financial information transparency are important paths to improve the comparability of accounting information. Therefore, enterprises should reasonably guide managers to reduce agency problems, which is the key enterprises’ benign development. In addition, our research helps to improve the comparability of corporate accounting information, thereby safeguarding the interests of stakeholders such as investors and creditors, and improving the efficiency of investor decision-making. (line 125-160)

**We believe that the suggestions you concerned will help to improve the quality of the section of contribution.**

**Comments 6:**

I barely understand several sentences, so I recommend to do proofread.

**Response 6:**

We are very sorry for the mis phrased sentences. According to your opinions, we have proofread the full paper in the hope that it will help readers understand better.

**Finally, thank you for your helpful suggestions. We sincerely hope that we have addressed all concerns in the revision. We look forward to continuing to improve our manuscript with your help.**

1. https://baijiahao.baidu.com/s?id=1619549428271560655&wfr=spider&for=pc
2. https://m.thepaper.cn/baijiahao_9396240
3. https://www.cs.com.cn/sylm/jsbd/201805/t20180518_5806392.html?open_source=weibo_search
